# The Maize NBS-LRR Gene *ZmNBS25* Enhances Disease Resistance in Rice and Arabidopsis

**DOI:** 10.3389/fpls.2018.01033

**Published:** 2018-07-17

**Authors:** Yunjian Xu, Fang Liu, Suwen Zhu, Xiaoyu Li

**Affiliations:** ^1^School of Life Sciences, Anhui Agricultural University, Hefei, China; ^2^National Engineering Laboratory of Crop Stress Resistance Breeding, Anhui Agricultural University, Hefei, China; ^3^College of Agronomy, Anhui Agricultural University, Hefei, China

**Keywords:** disease resistance, NBS-LRR, maize, rice, Arabidopsis

## Abstract

Nucleotide-binding site-leucine-rich repeat (NBS-LRR) domain proteins are immune sensors and play critical roles in plant disease resistance. In this study, we cloned and characterized a novel NBS-LRR gene *ZmNBS25* in maize. We found that *ZmNBS25* could response to pathogen inoculation and salicylic acid (SA) treatment in maize, and transient overexpression of *ZmNBS25* induced a hypersensitive response in tobacco. High-performance liquid chromatography (HPLC) analysis showed that, compared to control plants, *ZmNBS25* overexpression (*ZmNBS25*-OE) in Arabidopsis and rice resulted in higher SA levels. By triggering the expression of certain defense-responsive genes, *ZmNBS25*-OE enhanced the resistance of Arabidopsis and rice to *Pseudomonas syringae* pv. *tomato* DC3000 and sheath blight disease, respectively. Moreover, we found little change of grain size and 1000-grain weight between *ZmNBS25*-OE rice lines and controls. Together, our results suggest that *ZmNBS25* can function as a disease resistance gene across different species, being a valuable candidate for engineering resistance in breeding programs.

## Introduction

Plants have evolved multiple defense strategies against pathogen infections (Thomma et al., 2011; Henry et al., 2013) and have different immune systems that are highly effective against most microbial pathogens (Jones and Dangl, 2006; Chen and Ronald, 2011; Dangl et al., 2013). The first defense system recognizes conserved pathogen-associated molecular patterns (PAMPs) and is called PAMP-triggered immunity. This first line of defense kills many pathogens, while the other systems suppress or bypass infection (Jones and Dangl, 2006; Zhang and Zhou, 2010; Chen and Ronald, 2011). The second defense system recognizes specific pathogen effectors, such as the avirulence protein (Avr), which is produced by pathogens in an attempt to suppress host defenses and cause disease. This process is called effector-triggered immunity (ETI) (Nurnberger et al., 2004) and relies on the specific recognition of pathogen effectors by disease resistance (R) proteins. The rapid defense reaction initiated by ETI, i.e., the hypersensitive response (HR) (Dangl et al., 1996; Greenberg, 1997), prevents the spread of microbial pathogens and infection in plants (Zvereva and Pooggin, 2012). It ultimately leads to systemic acquired resistance (SAR), which confers long-lasting protection against a broad spectrum of microorganisms (Chen and Ronald, 2011; Molinari, 2016).

R proteins can act as receptors that directly or indirectly recognize the Avr and form R-Avr complexes to activate various resistance responses (Liu et al., 2007; Cesari et al., 2013; Sohn et al., 2014). Most reported plant *R* genes belong to the nucleotide-binding site-leucine-rich repeat (NBS-LRR) gene family (Jones and Dangl, 2006) and comprise C-terminal leucine-rich repeat (LRR) and central nucleotide-binding site (NBS) domains. In most cases, a Toll/interleukin-1 receptor (TIR) domain homology region or coiled-coil (CC) domain is located at their N-terminal, and these genes are called TIR-NBS-LRR and CC-NBS-LRR genes, respectively (Takken and Goverse, 2012). Plant NBS-LRR genes interact with pathogen effector proteins to activate signal transduction pathways involved in innate immunity while TIR and CC domains specifically recognize R-Avr complexes and initiate downstream defense signaling (Burkhard et al., 2001; Liu et al., 2007). The LRR domain may determine resistance specificity and be primarily responsible for the recognition of R-Avr complexes (Jones and Dangl, 2006; Liu et al., 2007).

The functions of NBS-LRR genes have been studied in several species. For example, *ZmRXO1* isolated from maize (*Zea mays* L.) is involved in transformed rice (*Oryza sativa* L.) resistance against *Xanthomonas oryzae* pv. *oryzicola*, which causes rice blast, revealing the feasibility of non-host *R* gene transfer between crops (Zhao et al., 2005). *AhRRS5*, a novel NBS-LRR resistance gene from peanut (*Arachis hypogaea* L.), was up-regulated in response to *Ralstonia solanacearum*, and its transient overexpression in *Nicotiana benthamiana* leaves induces HR (Zhang et al., 2017). Overexpression of *AhRRS5* in tobacco (*N. tabacum* L.) significantly enhanced its resistance to *R. solanacearum* (Zhang et al., 2017). Heterologous overexpression of the *Prunus sogdiana* Vassilcz NBS-LRR gene *PsoRPM2* in tobacco enhanced its resistance to root-knot nematode (*Meloidogyne incognita*) infection (Zhu et al., 2017), and overexpression of the grapevine *Vitis amurensis* Rupr. TIR-NBS-LRR gene *VaRGA1* in tobacco enhanced its resistance to *Phytophthora parasitica* (Li et al., 2016). In addition, the expression of NBS-LRR genes is always correlated with pathogen infection or salicylic acid (SA) treatment. For instance, the Arabidopsis *RPP2* gene, which includes the *RPP2A* and *RPP2B* isoforms, confers downy mildew resistance and is required for SA-dependent ETI (Sinapidou et al., 2004; Bonardi et al., 2011).

Maize is an important crop grown worldwide and it is susceptible to many diseases that can significantly lower its yield. For example, southern leaf blight (SLB) caused by the filamentous ascomycete *Bipolaris maydis* is widespread and has high infectivity in maize (Shurtleff, 1980). Host plants are infected by asexual spores (conidia) of *B. maydis* through wind and rain (Sumita et al., 2017). It is estimated that, in 1970, an SLB epidemic caused 15% drop in total maize production and a loss of one billion dollars (Ullstrup, 1972). Therefore, a better understanding of the NBS-LRR gene in maize might enable predicting and identifying genes that play important roles in heterologous systems and such knowledge might be used to greatly improve the stability of agricultural system. In our previous study, we identified 109 NBS-encoding genes based on the complete genome sequence of maize and found that some of these genes responded to *B. maydis* infection (Cheng et al., 2012). The phylogenetic analysis of the 109 NBS-encoding gene sequences revealed that some maize NBS-encoding genes shared high similarity to NBS-encoding genes with known functions. *ZmNBS25* was included in this set of NBS-encoding genes and clustered with *Arabidopsis thaliana* L. *AtRPM1* in the phylogenetic tree (Cheng et al., 2012). However, the response of *ZmNBS25* to *B. maydis* or SA and its functions are yet to be elucidated. In the present study, we isolated the NBS-LRR gene *ZmNBS25* from maize and investigated its disease resistance functions in Arabidopsis and rice. Our results indicate that *ZmNBS25* plays important roles and can be functional across different species against diverse pathogens. Thus, it might be a valuable candidate for engineering pathogen resistance in breeding programs.

## Materials and Methods

### Maize Materials and Treatments

Maize CMT030 plants were grown in a greenhouse at 28°C under a 16 h light/8 h dark cycle. The fungal strain was grown at 28°C in potato dextrose agar (PDA) medium for 7 days. Healthy maize seedlings grown for 14 days (3-leaf stage) were treated with a *B. maydis* spores suspension (10^5^ mL^−1^) in sterile deionized water. The *B. maydis* spore suspension was sprayed onto maize leaves, and these were covered with plastic film for 24 h to maintain moisture in the treated areas. For SA treatment, 1 mM SA (Sangon Biotech Co., Ltd., Shanghai, China) was sprayed onto maize seedlings not subject to *B. maydis* infection. The leaves were harvested for RNA extraction at 0, 12, 24, 48, and 60 h post *B. maydis* inoculation, and at 0, 1, 6, 12, and 24 h post SA treatment. Untreated plants were harvested at the same time points and used as controls. Six maize plants were collected per treatment at each time point. Three biological replicates were used for all treatments.

### Bioinformatics Analysis

The GSDS website (Gene Structure Display Server^1^) was used to analyze *ZmNBS25* gene structure (Zhang et al., 2013). Alignments between ZmNBS25 and other functional R proteins were performed in MEGA6 (Koichiro et al., 2013), and a phylogenetic tree was constructed by the neighbor-joining method based on whole protein sequences and considering 1,000 bootstrap replicates (Liu et al., 2016). Spatio-temporal expression of *ZmNBS25* during maize development was investigated using the microarray data of B73 maize from PLEXdb (Sekhon et al., 2011; Dash et al., 2012). A heat map of the spatio-temporal expression of *ZmNBS25* was generated in R/Bioconductor^2^. Promoters of *ZmNBS25* were analyzed by RSAT^3^ (Medina-Rivera et al., 2015).

### Full-Length cDNA Cloning

Total RNA extracted from the leaves of CMT030 seedlings was used to synthesize first-strand cDNA. The full-length cDNA products of *ZmNBS25* were obtained using PrimeSTAR Max DNA Polymerase (TaKaRa Bio Inc., Kusatsu, Shiga, Japan) and forward (5′-ATGGCAGAAGCTGTGGTGTT-3′) and reverse (5′-CTATATGCGCAACTCCAGACC-3′) primers, under 35 cycles of 98°C for 10 s, 55°C for 15 s, and 72°C for 15 s. The products were then cloned and sequenced.

### Vector Construction and Cell Death Assays in *N. benthamiana*

The full-length coding sequence of *ZmNBS25* without a termination codon was inserted into the vector pCAMBIA1301 to generate the 35S::*ZmNBS25* vector. The 35S::*ZmNBS25* and pCAMBIA1301 (control) vectors were transformed into the *Agrobacterium tumefaciens* strain GV3101. About 100 μL of *A. tumefaciens* suspensions carrying 35S::*ZmNBS25* or control constructs were injected into 4-week *N. benthamiana* leaves using a small syringe as previously described (Cesari et al., 2014; Zhang et al., 2017). Staining with 1 g L^−1^ 3′-Diaminobenzidine (DAB; Sigma-Aldrich, St. Louis, MO, United States) and 1 g L^−1^ lactophenol-trypan blue (Sangon Biotech Co., Ltd., Shanghai, China) was performed as previously described (Hwang and Hwang, 2011; Zhang et al., 2017). *N. benthamiana* leaves were treated overnight with DAB and the stained leaves were cleared with 95% ethanol. For trypan blue staining, leaves were boiled in the trypan blue solution for 5 min and destained overnight in 2.5 g mL^−1^ chloral hydrate. The destained leaves were observed under a microscope (Leica DM5000 B, Leica Microsystems Ltd., Heerbrugg, Switzerland). Three biological replicates were used for the experiment.

### Measurement of Electrolyte Leakage

Electrolyte leakage was determined according to Hwang and Hwang (2011). *N. benthamiana* leaves were infiltrated with *A. tumefaciens* GV3101 carrying 35S::*ZmNBS25* or control constructs to determine electrolyte leakage. Thirty *N. benthamiana* leaf disks approximately 1 cm in diameter were punched out with a cork borer, washed in 20 mL double distilled water for 30 min, and then transferred to fresh double distilled water (20 mL). Ion leakage was measured at 0 and 48 h after infiltration. Conductance was measured with a conductivity meter (DDS-11A) and the electrical conductivities of the media were expressed as μS cm^−1^. Three biological replicates were set for the experiment.

### Rice Transformation

The rice cultivar Zhonghua 11 was selected for *ZmNBS25* transformation. Embryogenic calli were obtained using 14-day-old immature embryos. Active embryogenic calli were harvested for transformation with *A. tumefaciens* GV3101 containing the 35S::*ZmNBS25* constructs, which was performed as previously described (Datta et al., 2000). Briefly, embryogenic calli were co-cultivated for 2 days with *A. tumefaciens* GV3101 in N6 medium (Sigma-Aldrich) containing 200 μM acetosyringone (Sangon Biotech Co., Ltd.). They were then thoroughly washed with sterile water and transferred to fresh medium containing 250 mg L^−1^ cefotaxime and 50 mg L^−1^ hygromycin (both Sangon Biotech Co., Ltd.). After several selection cycles, the embryogenic calli were used to propagate plants in regeneration medium. The transgenic plants were transplanted to pots filled with soil. Independent transgenic lines expressing 35S::*ZmNBS25* were identified by Southern blot analysis as previously described (Jia et al., 2011; Zhao et al., 2014). Genomic DNA was extracted from the leaves of T0 transgenic lines using CTAB method (cetyl trimethyl ammonium bromide) (Clarke, 2009), and digested by the restriction enzyme *EcoRV* (TaKaRa Bio Inc., Kusatsu, Shiga, Japan) overnight at 37°C. A fragment of the *hygromycin* gene, labeled with digoxigenin by a PCR DIG Probe Synthesis Kit (Roche Molecular Diagnostics, Pleasanton, CA, United States) was used as the hybridization probe.

### Arabidopsis Transformation

Arabidopsis plants were transformed with the floral dip method (Clough and Bent, 1998) using *A. tumefaciens* GV3101 carrying the 35S::*ZmNBS25* construct or the control vector. Transformed Arabidopsis seeds were sown onto Murashige and Skoog (MS; Sigma-Aldrich) agar plates containing 20 mg mL^−1^ hygromycin (Roche Molecular Diagnostics) to screen for positive transformants, which were then transplanted for further growth. Genomic DNA was extracted from the leaves of transgenic Arabidopsis lines using the CTAB (cetyl trimethyl ammonium bromide) method (Clarke, 2009). The DNA extracted from each transgenic Arabidopsis plant was used as template to determine positive transgene integration by PCR. The 2× Taq Master Mix (Dye Plus; Vazyme Biotech Co., Ltd., Nanjing City, PRC) was used for PCR amplification under the following profile: 95°C for 10 s, 32 cycles of 55°C for 30 s, and 72°C for 30 s. Seeds from positive transformants were harvested, subjected to hygromycin screening, and the process was repeated until only seeds capable of growing in hygromycin medium remained. The homozygous T3 generation was selected for subsequent experiments.

### Measurement of SA

In Arabidopsis and rice, SA was extracted and quantified according to a previously described method (Meuwly and Metraux, 1993; Cho et al., 2013). Briefly, leaves were flash-frozen in liquid nitrogen and ground to a very fine powder. The SA extracted from 0.5 g Arabidopsis leaf powder was determined by high-performance liquid chromatography (HPLC) (Agilent 1100 series with a C18 column, Agilent Technologies, Santa Clara, CA, United States), using SA from Dikma (Beijing, China) as the internal standard. The SA extracted from 0.3 g rice leaf powder was also determined by HPLC (Agilent 1200 series with a C18 column, Agilent Technologies) using SA from Sigma-Aldrich as the internal standard.

### Arabidopsis Pathogen Inoculation and Disease Index

*Pseudomonas syringae* pv. *tomato* DC3000 (*Pst* DC3000) was grown for 2 days at 28°C on King’s B (KB) medium (tryptone 20 g L^−1^, glycerol 10 mg L^−1^, K_2_HPO_4_ 1.5 g L^−1^, MgSO_4_ 1.5 g L^−1^, and agar 15 g L^−1^). After this period, a single *Pst* DC3000 colony was used to inoculate 5 mL KB medium and left to proliferate for 1 day at 28°C. One milliliter of the culture was used to inoculate 100 mL KB and left to grow for 1 day. The bacterial suspension was centrifuged at 3,000 rpm for 15 min and the pellet was homogenized in a resuspension solution of 0.01% Silwet L-77 and 10 mM MgSO_4_. The final optical density at 600 nm (OD_600_) was adjusted to 1.0 and Arabidopsis presenting 14 to 18 rosette leaves were sprayed with the bacterial suspension [10^5^ colony forming units (CFU) mL^−1^] and control were sprayed with resuspension solution without *Pst* DC3000 (mock). Plants were then covered with plastic film for 3 days to retain moisture in the treated area. Evaluation of the disease index (DI) was performed 7 days post inoculation (dpi) as follows: DI (%) = [Σ(ni × vi)/(V × N)/100], where vi = disease rating; ni = number of plants with that disease rating; V = highest disease rating; and N = total number of observed plants (Niu et al., 2011). Three biological replicates were set and each replicate contained nine Arabidopsis plants. Cell death was measured by Evans blue staining as previously described (Xie et al., 2017).

### Rice Pathogen Inoculation

To evaluate the resistance of transgenic rice to disease, rice overexpressing *ZmNBS25* (*ZmNBS25*-OE) and control rice (EV) were inoculated with *Rhizoctonia solani* at the seedling stage using the leaf sheath inoculation method. *Rhizoctonia solani* cultured on PDA plates was cut into equal size pieces and then inoculated in rice leaf sheath (*ZmNBS25*-OE transgenic and control plant). Plants were then covered with plastic film to maintain moisture. Disease phenotype was obtained at 14 days post *R. solani* inoculation.

### Quantitative Real-Time Polymerase Chain Reaction (qRT-PCR) Analysis

Total RNA was isolated with TRIzol (Thermo Fisher Scientific, Waltham, MA, United States) from 6 maize plants, 12 Arabidopsis plants, and 6 rice plants subject to each treatment including maize treatment with SA, *B. maydis* and control, Arabidopsis treatment with *Pst*. DC3000 and control, rice treatment with *Rhizoctonia solani* and control. Three biological repeats were performed for each treatment. DNase (TaKaRa Bio Inc., Kusatsu, Shiga, Japan) was used to eliminate genomic DNA contamination. A reverse transcription kit (Roche Molecular Systems, Inc., Pleasanton, CA, United States) was used to synthesize first-strand cDNA from 1 μg total RNA from each sample. Quantitative real-time polymerase chain reactions (qRT-PCR) were run on an Applied Biosystems 7300 system (Applied Biosystems, Foster City, CA, United States) using the primers listed in **Supplementary Table S1**. The relative expression level of genes investigated in this study was calculated with the formula 2^−ΔΔCt^, ΔΔCt = (CT_gene_ - CT_actin_)_treat_ - (CT_gene_ - CT_actin_)_control_ (Zhang et al., 2017).

### Statistical Analyses

The data were analyzed by Student’s *t*-test to evaluate differences between control and treated samples. Statistical significance was set at ^∗^*P* < 0.05, ^∗∗^*P* < 0.01, ^∗∗∗^*P* < 0.001. Each assay contained three independent replicates.

## Results

### Characterization of *ZmNBS25*

**FIGURE 1:**
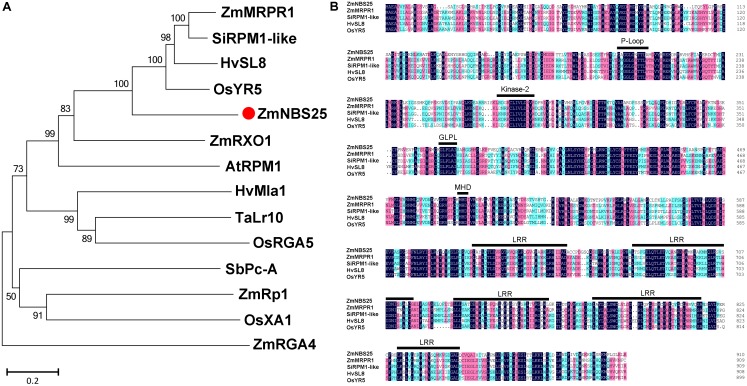
Comparison of ZmNBS25 with other disease resistance proteins. **(A)** Phylogenetic analysis of ZmNBS25 and other plant resistance proteins. Bootstrap values (1,000 replicates) are shown as percentages at the branch nodes. Bar = 0.2. GenBank accession numbers and plant sources are: AtRPM1 (CAA61131.1) from *Arabidopsis thaliana*, HvSL8 (AJ507098) and HvMla1 (AAG37356.1) from *Hordeum vulgare*, OsXA1 (BAA25068.1), OsRGA5 (AGM61351.1), and OsYR5 (AF456245.1) from *Oryza sativa*, SbPc-A (ACB72454.1) from *Sorghum bicolor*, SiRPM1-like (XM_004978992.1) from *Setaria italica*, TaLr10 (ADM65840.1) from *Triticum dicoccoides*, ZmMRPR1 (NM_001112339.1), ZmRp1 (AAP81261.1), ZmRXO1 (AAX31149.1), and ZmRGA4 (NP_001147651.1) from *Zea mays*. **(B)** Conserved domain comparisons between the amino acid sequences of ZmNBS25 and other resistance proteins. Black, red, and blue shading represent 100%, ≥75%, and ≥50% amino acid sequence similarity, respectively.

Our previous study has identified 109 NBS encoding genes in maize (Cheng et al., 2012), including *ZmNBS25*. The protein encoded by this gene clustered with the Arabidopsis disease resistance protein AtRPM1 (Cheng et al., 2012). The full-length cDNA sequence of *ZmNBS25* (GRMZM2G050959) is 3077 bp long, containing a 2736 bp coding sequence (**Supplementary Figure S1**). The phylogenetic analysis, based on whole protein sequences, showed that ZmNBS25 clustered with HvSL8 of *Hordeum vulgare*, ZmMRPR1 of *Z. mays*, SiRPM1-like of *Setaria italica*, and OsYR5 of *O. sativa* (**Figure 1A** and **Supplementary Table S2**). Whole-protein sequence identities between ZmNBS25 and HvSL8, ZmMRPR1, SiRPM1-like, OsYR5 were 45.84, 45.54, 47.46, and 46.92%, respectively. Protein sequence motif analysis showed that ZmNBS25 has conserved NBS motifs, including P-Loop, GLPL, kinase-2, MHD motif, and a C-terminal LRR domain (Bertioli et al., 2003; Cheng et al., 2012; Cesari et al., 2014) (**Figure 1B**).

**FIGURE 2:**
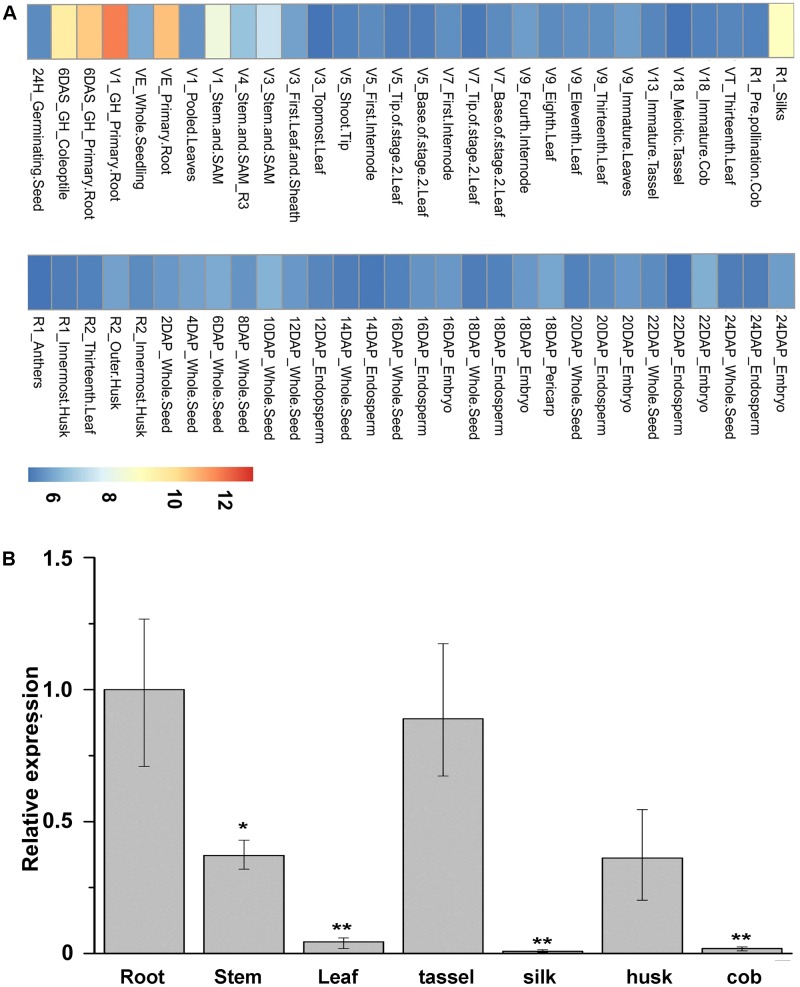
Spatial expression pattern of *ZmNBS25* in maize. **(A)** Heat map of *ZmNBS25* gene expression in maize. High, medium, and low expression levels are represented in red, yellow, and blue in the map, respectively. V, vegetative growth stage; R, reproductive growth stage; DAS, days after sowing, and DAP, days after pollination. **(B)** Expression pattern of *ZmNBS25* in different maize CMT030 tissues. Data represent mean relative expression values ± standard deviation from three independent experiments. The expression level in root was used as the control and assigned the value of 1. Asterisks indicate statistically significant differences between root and other tissues by Student’s *t*-test (^∗^*P*< 0.05, ^∗∗^*P*< 0.01).

Expression patterns of *ZmNBS25* in various maize organs and different developmental stages were investigated by using the microarray data. We found that *ZmNBS25* expressed highly in root and stem, but relatively low in other tissues, such as leaf and cob (**Figure 2A**). We further examined the spatial expression patterns of *ZmNBS25* in seven tissues (root, stem, leaf, tassel, silk, husk, and cob) of maize by qRT-PCR and found that, similar to the microarray analysis, *ZmNBS25* expression was high in root and stem, and low in leaf and cob (**Figure 2B**).

### *ZmNBS25* Is Induced in Leaf Upon *B. maydis* Infection and SA Treatment

**FIGURE 3:**
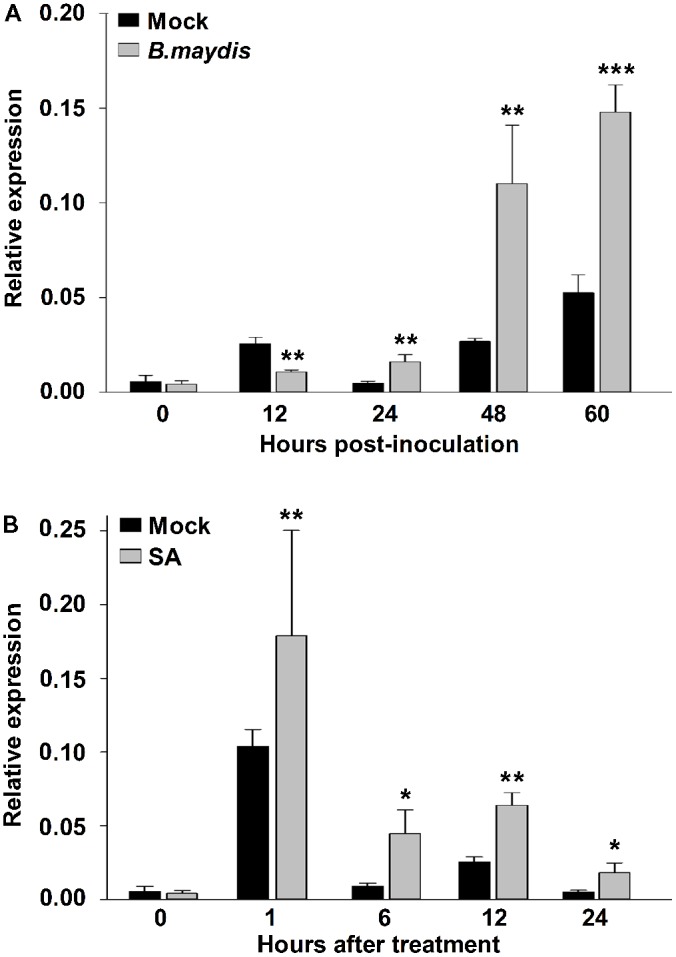
Gene expression analysis of *ZmNBS25* in maize leaf upon *Bipolaris maydis* inoculation and salicylic acid (SA) treatment. **(A)** Expression levels of *ZmNBS25* upon *B. maydis* inoculation. Maize seedlings at the 3-leaf stage were infected with *B. maydis*, and leaves were harvested at 0, 12, 24, 48, and 60 h post inoculation. **(B)** Expression levels of *ZmNBS25* treated with SA. Three-leaf stage maize seedlings received SA and leaves were harvested at 0, 1, 6, 12, and 24 h after SA treatment. Data were normalized using the transcript level of *ZmActin* and *Zmtubulin*, and relative expression levels of *ZmNBS25* at different time points are shown as folds of the level of *ZmActin*. Data represent mean relative expression values ± standard deviation from three independent experiments. Asterisks indicate statistically significant differences between treated and untreated maize by Student’s *t*-test (^∗^*P*< 0.05, ^∗∗^*P*< 0.01, ^∗∗∗^*P*< 0.001).

To test whether *ZmNBS25* is responsible for disease resistance in maize, we sprayed either a spore suspension of *B. maydis* or SA on leaves to mimic the natural disease environment. After inoculating maize CMT030 with *B. maydis*, the expression level of *ZmNBS25* was first reduced at 12 h post inoculation (hpi), followed by a significantly increased at 24, 48, and 60 hpi (**Figure 3A**). On the other hand, after SA treatment, the expression level of *ZmNBS25* was consistently increased from 1 to 24 hpi when compared to control samples (**Figure 3B**).

### Transient Overexpression of *ZmNBS25* in *N. benthamiana* Leaves Induces HR

**FIGURE 4:**
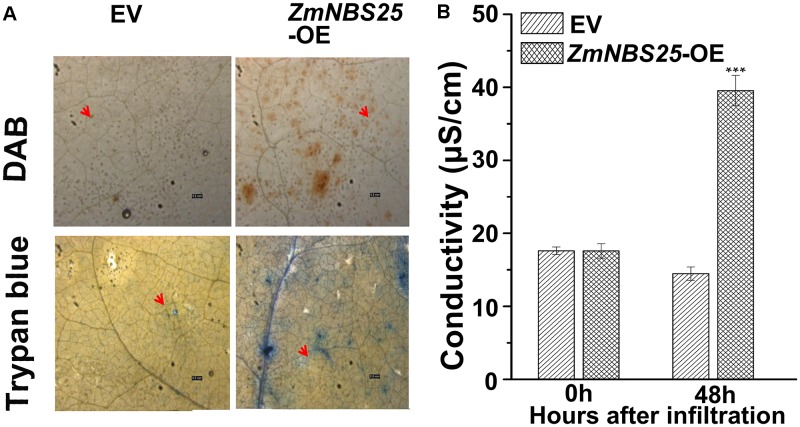
Effect of transient *ZmNBS25* expression on immunity induction in *Nicotiana benthamiana*. **(A)** DAB staining (above) and trypan blue staining (below) of *N. benthamiana* leaves that transiently express 35S::*ZmNBS25* and pCAMBIA1301. **(B)** Electrolyte leakage of *N. benthamiana* leaves infiltrated with either 35S::*ZmNBS25* or pCAMBIA1301. Bars = 0.5 mm. The arrow indicates the infiltration spot. Data represent mean relative expression values ± standard deviation from three independent experiments. Asterisks indicate statistically significant differences between pCAMBIA1301 and 35S::*ZmNBS25* tobacco by Student’s *t*-test (^∗∗∗^*P*< 0.001).

To determine whether *ZmNBS25* is involved in disease resistance in other crops, we cloned *ZmNBS25* from maize CMT030 and generated an overexpression vector of *ZmNBS25* using the pCAMBIA1301 vector with a CaMV35S promoter. After transforming the plasmid in *A. tumefaciens* GV3101, this was infiltrated into *N. benthamiana* leaves to verify whether transient *ZmNBS25* overexpression could cause HR cell death. Large amounts of H_2_O_2_ accumulation were observed in *N. benthamiana* leaves after *ZmNBS25* transient overexpression for 48 h by DAB staining (**Figure 4A** and **Supplementary Figure S2**). Trypan blue staining and electrical conductivity are two well established indicators of electrolyte leakage and correlate with the severity of visual damage (Zhang et al., 2016). As shown in **Figure 4B**, ion conductivity significantly increased in plants overexpressing *ZmNBS25* compared with plants expressing pCAMBIA1301. Furthermore, trypan blue staining was darker in the leaves of *ZmNBS25*-OE plants than in the leaves of pCAMBIA1301 transgenic plants (**Figure 4A** and **Supplementary Figure S2**). These results suggested that the transient overexpression of *ZmNBS25* in tobacco leaves induced HR and H_2_O_2_ accumulation, acting as a defense response to stress.

### *ZmNBS25* Overexpression in Arabidopsis Enhances Resistance to *Pst* DC3000

**FIGURE 5:**
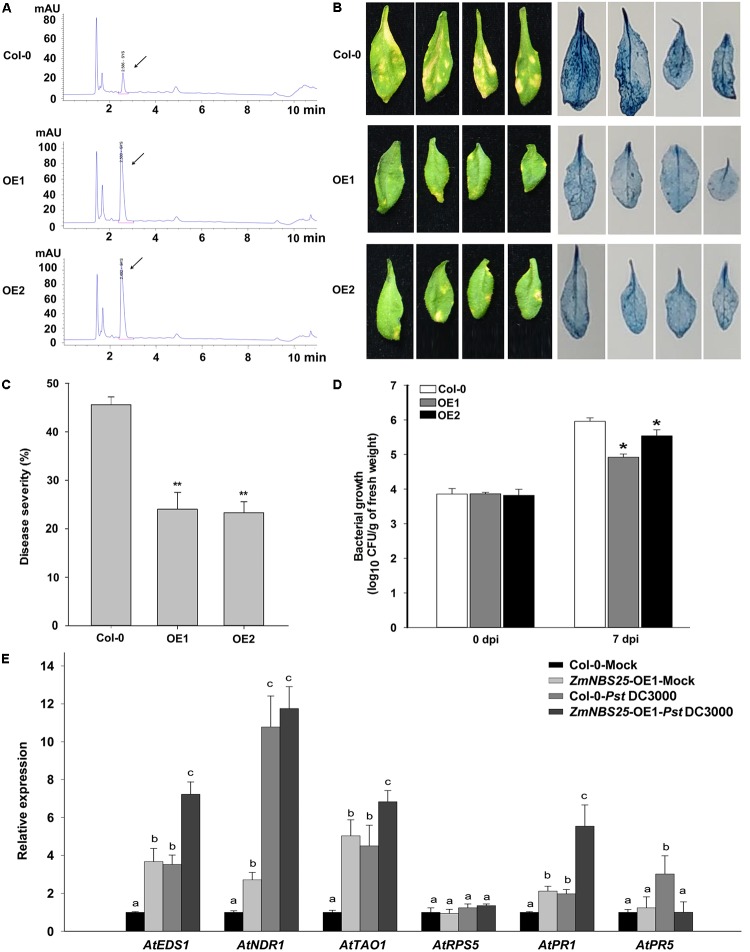
Enhanced resistance of *ZmNBS25*-OE transgenic Arabidopsis plants to *Pst* DC3000. **(A)** Chromatogram of SA extracted from *ZmNBS25*-OE and Col-0 plants without pathogen inoculation. mAU indicates peak height. The arrow indicates the SA peak. **(B)** Symptoms and Evans blue staining of *ZmNBS25*-OE and Col-0 leaves after 7 days of *Pst* DC3000 infection. Blue areas represent cell death. **(C)** Disease index of *ZmNBS25*-OE and Col-0 plants upon *Pst* DC3000 infection. **(D)** *Pst* DC3000 density on the infected leaves of *ZmNBS25*-OE and Col-0 plants. Leaf samples were collected at 0 and 7 days post inoculation (dpi). CFU represents colony-forming unit. **(E)** Expression levels of defense related genes in *ZmNBS25*-OE and Col-0 plants with or without *Pst* DC3000 infection. OE1 and OE2 represent two different transgenic lines of *ZmNBS25-*overexpressed Arabidopsis. **(C,D)** Asterisks indicate statistically significant differences between *ZmNBS25*-OE lines and Col-0 plants by Student’s *t*-test (^∗^*P*< 0.05, ^∗∗^*P*< 0.01). **(E)** Data were normalized using the transcript level of *AtActin* and *AtUbiquitin*. The expression levels of genes in Col-0 plants without *Pst* DC3000 infection were used as controls and assigned the value of 1. Different letters above the columns indicate significant differences at *P*< 0.05 level among Col-0-Mock, *ZmNBS25*-OE1-Mock, Col-0-*Pst* DC3000, and *ZmNBS25*-OE1-*Pst* DC3000. Data represent mean ± standard deviation from three independent experiments.

To investigate whether *ZmNBS25* has a universal role in disease resistance, we cloned *ZmNBS25* into a pCAMBIA1301 binary vector and generated *ZmNBS25-*OE transgenic Arabidopsis plants (**Supplementary Figure S3**). As shown in the HPLC chromatogram, total SA levels were drastically increased in *ZmNBS25*-OE plants (Peak area of OE1 was 460.4 g^−1^ and peak area of OE2 was 486.2 g^−1^) compared with non-transgenic Col-0 plants (Peak area was 75.5 g^−1^) without any treatment (**Figure 5A**). We then examined the effect of *ZmNBS25* overexpression after *Pst* DC3000 inoculation. Two transgenic Arabidopsis lines and Col-0 were sprayed with a virulent *Pst* DC3000 suspension (10^5^ CFU mL^−1^). Seven days after inoculation, the *ZmNBS25*-OE lines showed significant resistance to *Pst* DC3000 (**Figures 5B–D**). Evans blue staining of infected Arabidopsis plants showed that cell death was more evident in Col-0 plants than in *ZmNBS25*-OE plants (**Figure 5B**). The disease severity of *ZmNBS25*-OE lines was 23.66% on average, while that of Col-0 plants was 45.58% (**Figure 5C**). The density of *Pst* DC3000 on *ZmNBS25*-OE plants was significantly lower than in Col-0 plants at 7 dpi (**Figure 5D**). Moreover, the density of *Pst* DC3000 grown on the *ZmNBS25*-OE1 plants were significant lower than that on the *ZmNBS25*-OE2 plants at 7 dpi (*P* = 0.0114) (**Figure 5D**). This might be caused by the higher *ZmNBS25* expression in *ZmNBS25*-OE1 Arabidopsis plants than in *ZmNBS25*-OE2 plants (**Supplementary Figure S3**). These results strongly suggest that *ZmNBS25* plays important roles in disease resistance.

To examine if the enhanced resistance to *Pst* DC3000 is related to changes in defense responsive genes, we measured the expression levels of some defense related genes in Col-0 and *ZmNBS25*-OE plants upon *Pst* DC3000 infection. Particularly, we examined the relative expression levels of genes involved in pathogen resistance (PR) and on the SA-dependent defense signaling pathway, including *AtEDS1* (Enhanced Disease Susceptibility 1) (Falk et al., 1999), *AtNDR1* (Non-race Specific Disease Resistance 1) (Day et al., 2006), *AtTAO1* (Target of AvrB Operation) (Eitas et al., 2008), *AtRPS5* (Resistance to *P. syringae* protein 5) (Warren et al., 1998), *AtPR1*, and *AtPR5* in Col-0 and *ZmNBS25*-OE plants. Except for *AtRPS5* and *AtPR5*, defense genes showed higher expression levels in *ZmNBS25*-OE plants than in Col-0 plants without *Pst* DC3000 infection. On the other hand, the expression levels of *AtEDS1*, *AtNDR1, AtTAO1*, *AtPR1*, and *AtPR5* in Col-0 plants were significantly induced by *Pst* DC3000 (**Figure 5E** and **Supplementary Tables S3**, **S4**). Similar inductions were observed in *Pst* DC3000-infected *ZmNBS25*-OE plants compared with control *ZmNBS25*-OE plants, except *AtPR5*. When compared with *Pst* DC3000-infected Col-0 plants, the expression levels of *AtEDS1*, *AtTAO1*, and *AtPR1* were significantly increased in the *Pst* DC3000-infected *ZmNBS25*-OE plants (**Figure 5E** and **Supplementary Tables S3**, **S4**). These results indicate that *ZmNBS25* overexpression enhanced disease resistance of transgenic Arabidopsis against *Pst* DC3000.

### Transgenic Overexpression of *ZmNBS25* in Rice Confers Disease Resistance Without Affecting Grain Yield

**FIGURE 6:**
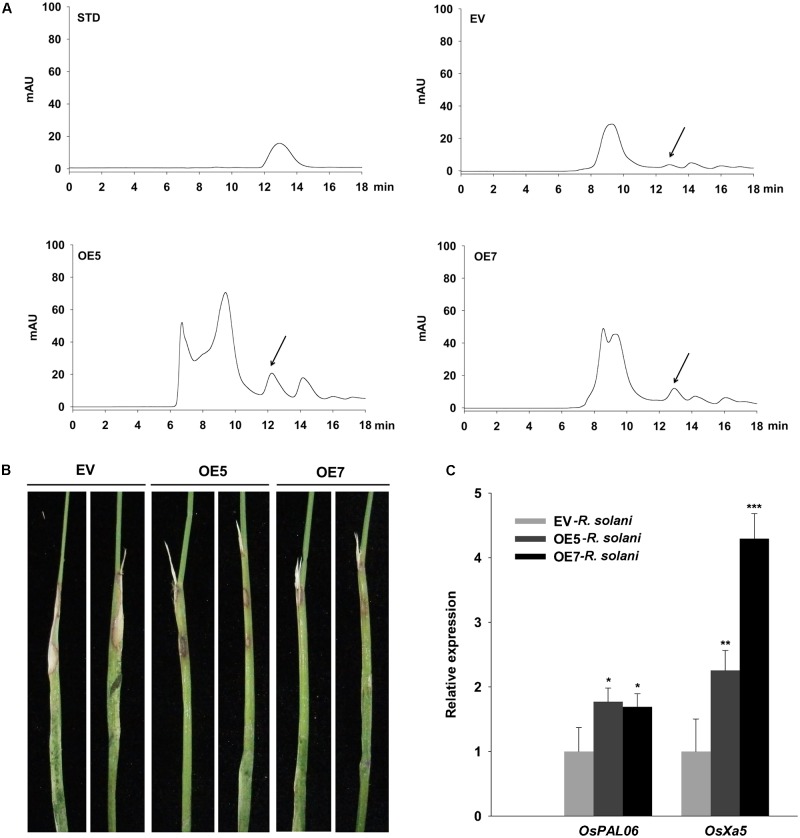
Functional analysis of the *ZmNBS25* gene in rice. **(A)** Chromatogram of SA extracted from *ZmNBS25*-OE and control (EV) rice plants without pathogen inoculation. STD represents standard SA; mAU indicates peak height. The arrow indicates the SA peak. **(B)** Resistance of *ZmNBS25*-OE plants to rice sheath blight. **(C)** Expression levels of defense related genes in *ZmNBS25*-OE and control plants inoculated with *Ralstonia solani*. Data were normalized using the transcript level of *Ostubulin* and *18srRNA*. OE5 and OE7 represent two different transgenic lines of *ZmNBS25-*overexpressed rice. The expression levels of genes in *R. solani*-infected EV plants were used as controls and assigned the value of 1. Asterisks indicate statistically significant differences between treated and untreated maize by Student’s *t*-test (^∗^*P*< 0.05, ^∗∗^*P*< 0.01, ^∗∗∗^*P*< 0.001). Data represent mean relative expression values ± standard deviation from three independent experiments.

To determine whether *ZmNBS25* overexpression confers disease resistance in other heterologous plant systems, we generated *ZmNBS25*-OE transgenic rice lines. Southern blot analysis showed three separate transfer (T)-DNA insertions events with only one copy each (1, 5, and 7; **Supplementary Figure S4**); thus, we selected two independent transgenic lines, L5 and L7, for subsequent experiments. Plant resistant against biotrophic pathogens is usually regulated by the SA-dependent pathway (Felton and Korth, 2000), so we first measured the SA content in *ZmNBS25*-OE and control plants. HPLC chromatograms showed that *ZmNBS25-*OE lines accumulated more SA (peak area 309.489 g^−1^) than control transgenic plants (peak area 74.749 g^−1^) without pathogen inoculation (**Figure 6A**). To further determine the disease resistance capacity of *ZmNBS25* overexpression, we inoculated *R. solani*, which can cause rice sheath blight, at rice leaf sheath. *ZmNBS25*-OE transgenic rice lines developed fewer and smaller disease lesions than control transgenic plants at 14 dpi (**Figure 6B**). We found that two typical defense related genes, *OsPAL06* and *OsPXa5*, were significantly upregulated in *ZmNBS25*-OE lines after *R. solani* infection (**Figure 6C**).

To investigate the effect of overexpressing the *ZmNBS25* gene on yield-related traits, we compared seeds’ size and 1000-grain weight between wild type (WT) and *ZmNBS25*-OE rice lines (**Supplementary Figure S5**). No phenotypic differences, including seed length and width, were observed between WT and *ZmNBS25*-OE rice lines (**Supplementary Figures S5A,C,D**). Except for *ZmNBS25*-OE line L7, no significant reductions in 1000-grain weight were observed in *ZmNBS25*-OE lines (L1, L5) (**Supplementary Figure S5B**). In addition, we observed similar results in Arabidopsis, where seeds showed no phenotypic differences between WT and *ZmNBS25*-OE lines (**Supplementary Figure S6**).

## Discussion

### *ZmNBS25* Is a Novel Maize NBS-LRR Resistance Gene

Nucleotide-binding site-leucine-rich repeat proteins play important roles in pathogen recognition and defense response signal transduction (Ameline-Torregrosa et al., 2008; Gao et al., 2010). Some NBS-LRRs that confer resistance to microbial pathogens and certain environmental stressors have been cloned from higher plants (Liu et al., 2007). In the present study, we cloned and systematically characterized a novel NBS-LRR-encoding gene, *ZmNBS25*, in maize. We found that *ZmNBS25* could be induced by *B. maydis* inoculation and SA treatment. Overexpression of *ZmNBS25* provides enhanced disease resistance in transgenic rice and Arabidopsis. This is similar to the function of *ZmRXO1*, which is a maize NBS-LRR gene involved in resistance to diverse pathogens, including rice blast (Zhao et al., 2004, 2005). Our phylogenetic analysis based on whole-protein sequences also showed that ZmNBS25 is closely related to ZmRXO1. In addition, ZmNBS25 has typical NBS-ARC, P-Loop, GLPL, kinase-2, MHD, and other conserved motifs similar to S-L8, MRPR1, RPM1-like, and YR5 proteins, suggesting that *ZmNBS25* can participate in maize pathogen interactions or defense responses.

### *ZmNBS25* Is Involved in Defense Responses to Biotic Stresses

Our results indicated that *ZmNBS25* was expressed at relatively low levels in uninfected maize leaves; however, it was significantly upregulated at 24 h post *B. maydis* inoculation, indicating that *ZmNBS25* might be associated with *B. maydis* resistance in maize. Similar expression patterns have been observed in other plants NBS-LRR genes, such as: *AhRRS5*, a *R. solanacearum* resistance gene in peanut (Zhang et al., 2017); *Xa1*, a bacterial resistance gene from rice (Yoshimura et al., 1998); and *SacMi*, a *M. incognita* resistance gene (Zhou et al., 2018). In addition, the expression level of *ZmNBS25* was significantly upregulated by SA treatment. SA is a well-known signaling molecule involved in defense against stress (Divi et al., 2010). It accumulates in plant tissues challenged by pathogen infections, and it can induce SAR and increase the expression of certain PR genes, which generally increase resistance to a wide range of diseases (Gaffney et al., 1993; Yang et al., 2013; Novakova et al., 2014). The expression of disease resistance genes such as *RCY1* and *NPR1* in Arabidopsis partially depends upon SA signaling (Cao et al., 1997; Takken and Joosten, 2000). Given the similar expression pattern of *ZmNBS25* upon SA treatment and *B. maydis* induction, the enhanced defense response to *B. maydis* in *ZmNBS25*-OE lines is likely associated with SA signaling. Moreover, we also found that binding elements such as WBOXATNPR1, WRKY71OS, BIHD1OS, and GCCCORE could participate in plant disease resistance and were enriched in the *ZmNBS25* promoter (**Supplementary Figure S7**). For example, WBOXATNPR1 recognizes the WRKY DNA binding proteins induced by SA (Eulgem et al., 2000), and WRKY71OS interacts with certain WRKY family members that play vital roles in plant disease resistance (Eulgem et al., 2000; Shimono et al., 2012). Together, these results suggest that *ZmNBS25* plays a vital role in the defense response to diseases.

### *ZmNBS25* Confers Disease Resistance in Heterologous Plant Systems

Activation of NBS-LRR proteins triggers programmed cell death and reactive oxygen species (ROS) production in plants (Andersson et al., 2006; Qi and Innes, 2013). Indeed, we found that *ZmNBS25* might positively regulate SA-dependent cell death and disease resistance during pathogen infection. Transient overexpression of *ZmNBS25* in *N. benthamiana* induced a HR and caused cell death and H_2_O_2_ accumulation without pathogenic infection. Therefore, *ZmNBS25* could be involved in ROS signaling pathways for disease resistance. An *R* gene that can be integrated into a heterologous cereal crop will significantly improve disease resistance strategies for that species. An earlier study on the *ZmRXO1* gene in rice showed that it is possible to transfer a NBS-LRR-type *R* gene to a distantly related cereal species (Zhao et al., 2005). Studies have demonstrated that SA is involved in defense responses mediated by plant NBS-LRR R proteins (Roberts et al., 2013). For instance, the expression of ADR1-L2D848V in Arabidopsis caused increased disease resistance and constitutively high SA levels (Roberts et al., 2013). In our study, *ZmNBS25* overexpression in transgenic Arabidopsis and rice produced more SA than in control plants and enhanced transgenic plants resistance to disease, indicating that *ZmNBS25* might participate in disease responses by inducing SA accumulation.

### *ZmNBS25*-Mediated Pathogen Resistance Is Concomitant With the Upregulation of Defense-Related Genes

Without *Pst* DC3000 infection, the expression of defense genes *AtEDS1*, *AtNDR1*, *AtTAO1*, and *AtPR1* was higher in *ZmNBS25*-OE plants than in Col-0 plants, indicating that *ZmNBS25* overexpression affected the expression of defense-related genes and resulted in enhanced resistance to disease. In *Pst* DC3000 infected *ZmNBS25*-OE transgenic Arabidopsis plants, disease severity was reduced, and *ZmNBS25*-OE also affected the expression of some defense genes after *Pst* DC3000 inoculation as defense genes were significantly upregulated in *ZmNBS25*-OE plants after *Pst* DC3000 infection. Moreover, *ZmNBS25*-OE rice plants showed similar changes of defense-related genes after *R. solani* infection. These data indicated that *ZmNBS25* overexpression could promote the activation of defense response in Arabidopsis and rice plants infected with *Pst* DC3000 and *R. solani*, respectively. Taken together, our results suggest that *ZmNBS25* can function as a positive regulator to prime defense response upon pathogen infection.

### Outlook

The results in our current study strongly suggest that *ZmNBS25* can function as a disease resistance gene across different species, being a valuable candidate for engineering resistance in breeding programs. Given the common difficulties of gene editing in maize, a cost-effective and high-efficient transformation method is desired in the future to further evaluate the disease resistance function of *ZmNBS25* in maize by manipulating its gene expression.

## Author Contributions

YX, FL, SZ and XL conceived the project. YX carried out the experiments and FL performed the statistical analysis. YX, FL, SZ and XL wrote the manuscript.

## Conflict of Interest Statement

The authors declare that the research was conducted in the absence of any commercial or financial relationships that could be construed as a potential conflict of interest.

## Footnotes

1 http://gsds.cbi.pku.edu.cn/

2 http://www.bioconductor.org/

3 http://floresta.eead.csic.es/rsat/

## Supplementary Material

FIGURE S1 Gene structure of *ZmNBS25*.

FIGURE S2 Trypan blue and DAB staining of *N. benthamiana* leaves transiently expressing 35S::*ZmNBS25* and pCAMBIA1301. Bar = 0.5 mm. The arrow indicates the infiltration spot.

FIGURE S3 Relative expression levels of *ZmNBS25* in *ZmNBS25*-OE1 and *ZmNBS25*-OE2 transgenic Arabidopsis lines.

FIGURE S4 Southern blot analysis of *ZmNBS25*-OE transgenic rice lines.

FIGURE S5 Phenotypes of grain yields of wild type (WT) and the *ZmNBS25*-OE rice lines. **(A)** Grain size comparison between WT and *ZmNBS25*-OE seeds.**(B)** 1000-grain weights of WT and *ZmNBS25*-OE seeds. **(C)** Grain lengths of WT and *ZmNBS25*-OE seeds. **(D)** Grain widths of WT and *ZmNBS25*-OE seeds. Scale bars = 5mm. Student’s *t*-tests were performed between WT and *ZmNBS25*-OE rice lines (^∗∗∗^*P* < 0.001).

FIGURE S6 Seed phenotypes of WT and *ZmNBS25*-OE Arabidopsis lines.

FIGURE S7 Analysis of regulatory elements in the predicted promoter of *ZmNBS25*. The predicted promoter sequence was 2 kb upstream the *ZmNBS25* gene.

TABLE S1 Primers used for qRT-PCR in this study.

TABLE S2 Disease resistance proteins in various plant species.

TABLE S3 The cycle threshold (Ct) of defense-related genes in Arabidopsis from qRT-PCR program for **Figure 5E**.

TABLE S4 Statistical analyses of genes relative expression levels for **Figure 5E**.
