# Supplementary figures and images for: The Maize NBS-LRR Gene ZmNBS25 Enhances Disease Resistance in Rice and Arabidopsis
Source: Front Plant Sci. 2018 Jul 17;9:1033. doi: 10.3389/fpls.2018.01033 (PMC6056734; doi:10.3389/fpls.2018.01033)

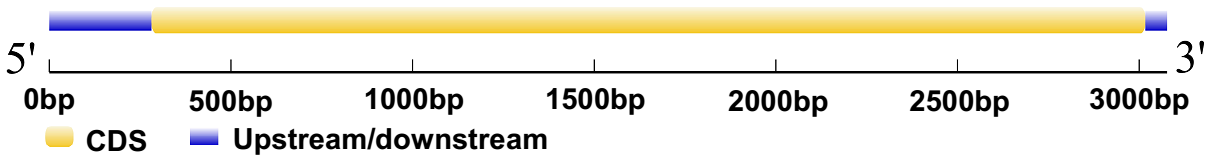

Supplement: FIGURE S1 — Gene structure of ZmNBS25. [file Image_1.TIF]

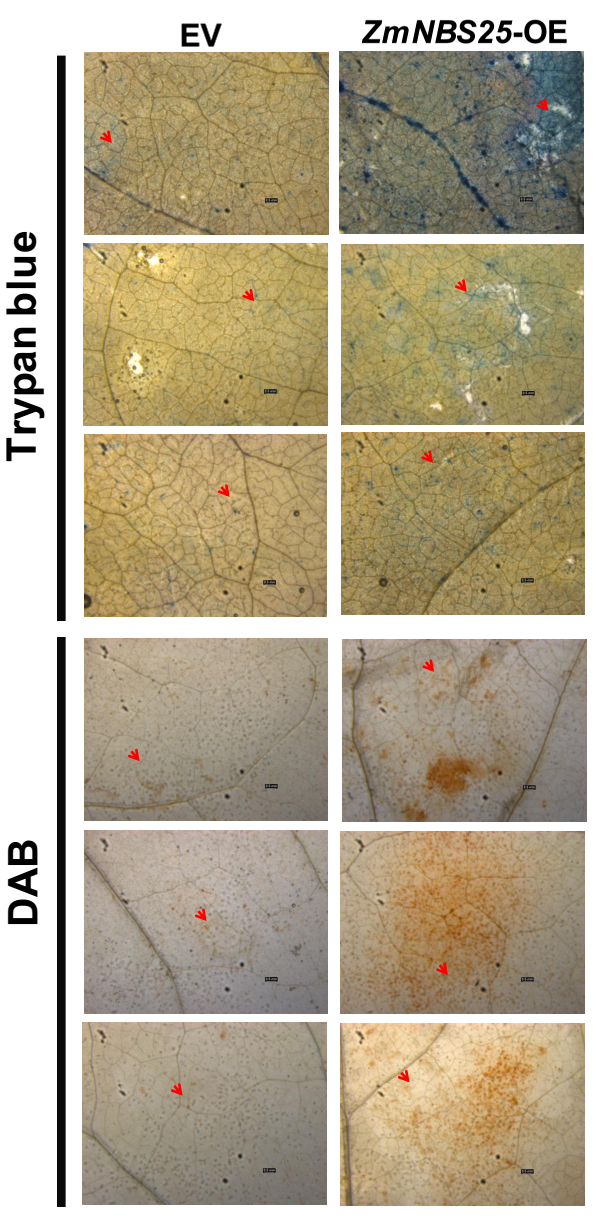

Supplement: FIGURE S2 — Trypan blue and DAB staining of N. benthamiana leaves transiently expressing 35S::ZmNBS25 and pCAMBIA1301. Bar = 0.5 mm. The arrow indicates the infiltration spot. [file Image_2.TIF]

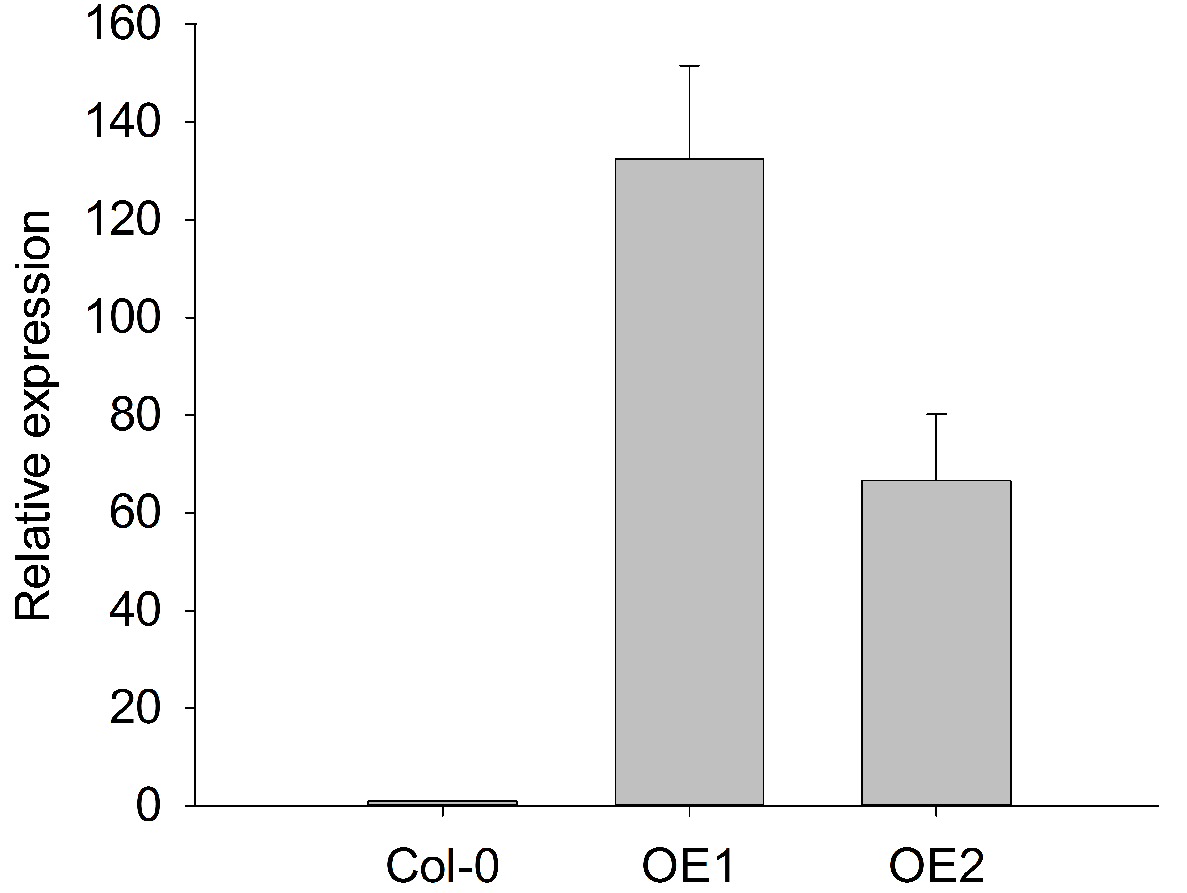

Supplement: FIGURE S3 — Relative expression levels of ZmNBS25 in ZmNBS25-OE1 and ZmNBS25-OE2 transgenic Arabidopsis lines. [file Image_3.TIF]

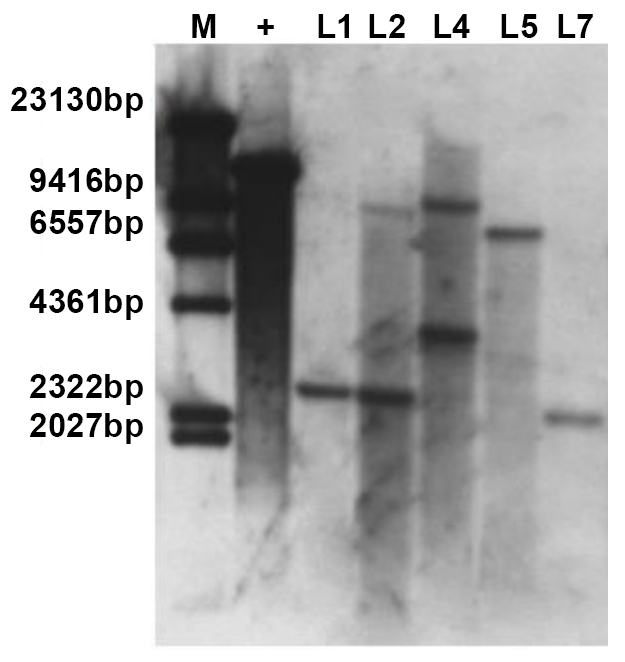

Supplement: FIGURE S4 — Southern blot analysis of ZmNBS25-OE transgenic rice lines. [file Image_4.TIF]

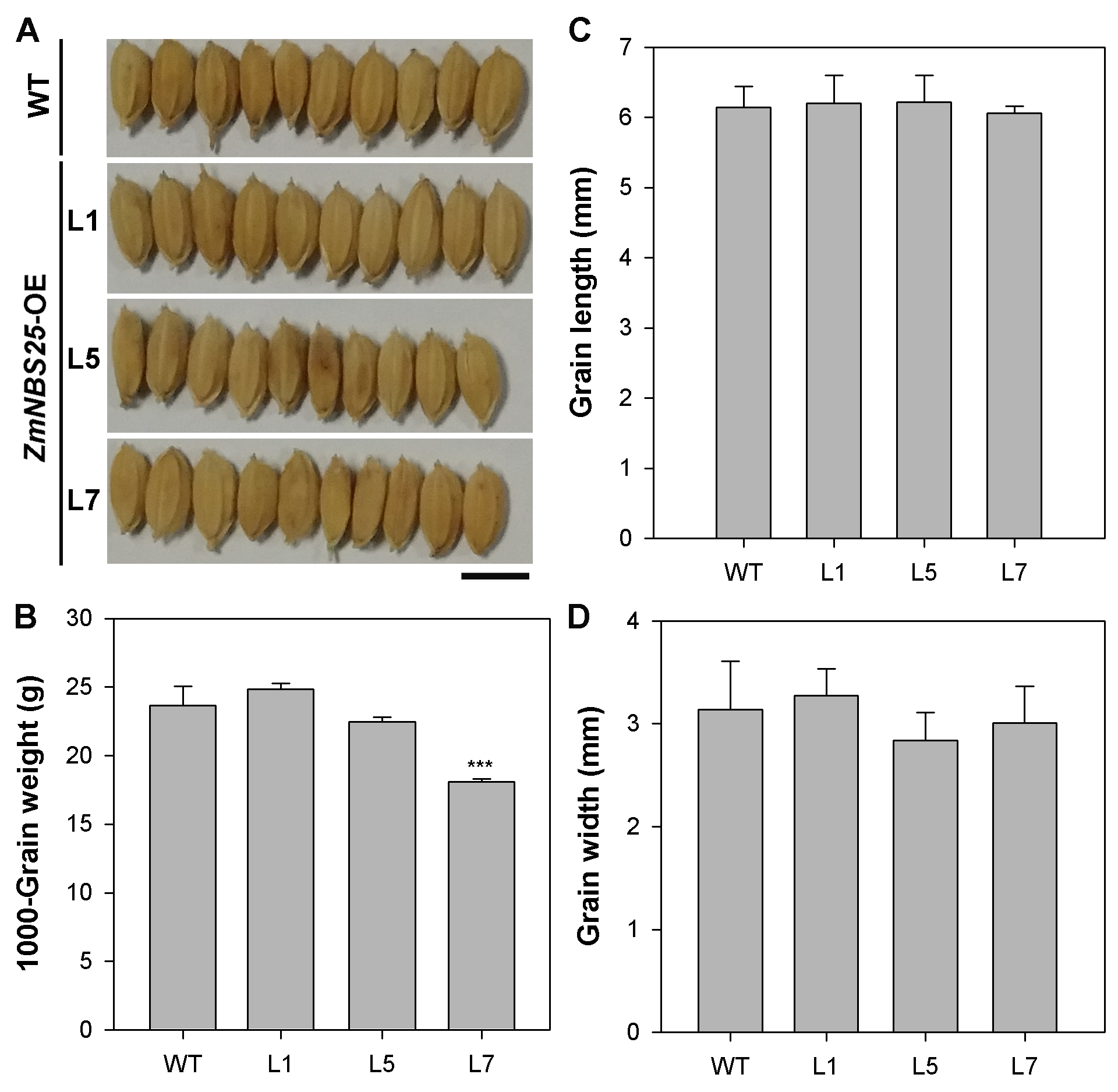

Supplement: FIGURE S5 — Phenotypes of grain yields of wild type (WT) and the ZmNBS25-OE rice lines. (A) Grain size comparison between WT and ZmNBS25-OE seeds.(B) 1000-grain weights of WT and ZmNBS25-OE seeds. (C) Grain lengths of WT and ZmNBS25-OE seeds. (D) Grain widths of WT and ZmNBS25-OE seeds. Scale bars = 5mm. Student’s t-tests were performed between WT and ZmNBS25-OE rice lines (∗∗∗P < 0.001). [file Image_5.TIF]

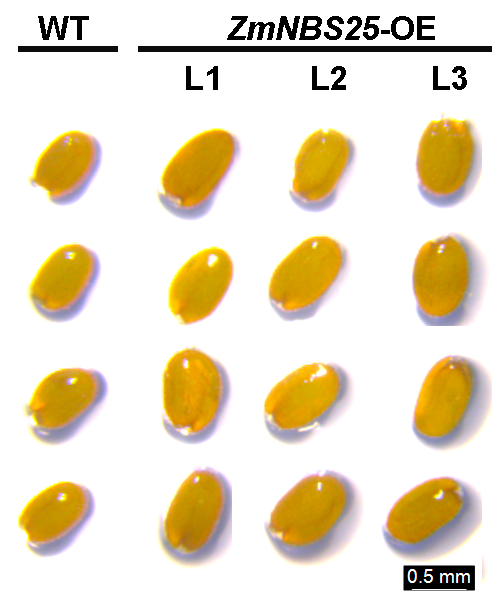

Supplement: FIGURE S6 — Seed phenotypes of WT and ZmNBS25-OE Arabidopsis lines. [file Image_6.TIF]

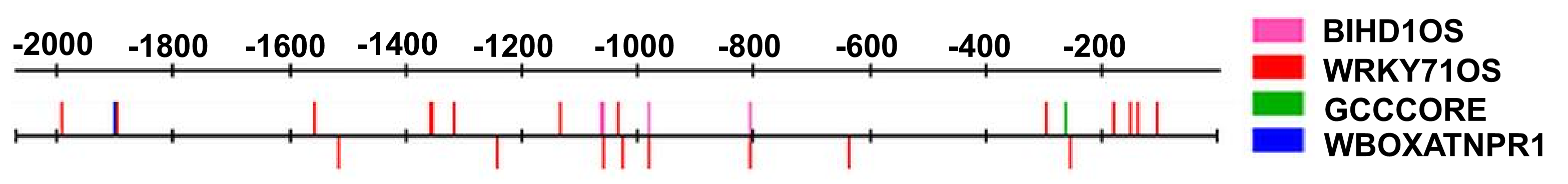

Supplement: FIGURE S7 — Analysis of regulatory elements in the predicted promoter of ZmNBS25. The predicted promoter sequence was 2 kb upstream the ZmNBS25 gene. [file Image_7.TIF]
