# Supplementary material for: The Maize NBS-LRR Gene ZmNBS25 Enhances Disease Resistance in Rice and Arabidopsis
Source: Front Plant Sci. 2018 Jul 17;9:1033. doi: 10.3389/fpls.2018.01033 (PMC6056734; doi:10.3389/fpls.2018.01033)
Supplement: TABLE S1 — Primers used for qRT-PCR in this study. [file Table_1.DOCX]

Table S1 Primers used for qRT-PCR in this study

| Primer names | Primer sequence |
| --- | --- |
| qNBS25_F | GTCTGCCATTTCCGGTCTGA |
| qNBS25_R | CCTCAACCTGGTAGGCCAAG |
| qZmActin_F | GGGATTGCCGATCGTATGAG |
| qZmActin_R | GAGCCACCGATCCAGACACT |
| qZmtubulin_F | GTTGGGAAGGAGATTGTCGAT |
| qZmtubulin_R | TGGCCTTTCGATGTCAAG |
| qAtEDS1_F | GAAGACCAACCCGCTACATATA |
| qAtEDS1_R | CTTTGAGTTCTTCAACCTCAGC |
| qAtNDR1_F | AGGTTGGATCTCAAGACTCAAG |
| qAtNDR1_R | CTTCAACATCAGCTCCAACTTC |
| qAtTAO1_F | TCAGAAGGGAAAGGATTGAGAC |
| qAtTAO1_R | AGTATCAACCAAGAACTGACGT |
| qAtRPS5_F | TCTCAAAGGTTGGTGGAATGTA |
| qAtRPS5_R | TGGCAAAGTCAATACTCTCACT |
| qAtPR1_F | CTAACTACAACTACGCTGCGAAC |
| qAtPR1_R | TTCATTAGTATGGCTTCTCGTTCA |
| qAtPR5_F | AGTTAGCTCCGGTACAAGTG |
| qAtPR5_R | GCTTAAGGTCATGGATCAGAAC |
| qAtUbiquitin_F | CTGCGACTCAGGGAATCTTCTAA |
| qAtUbiquitin_R | TTGTGCCATTGAATTGAACCC |
| qAtActin_F | GGCGATGAAGCTCAATCCAAACG |
| qAtActin_R | GGTCACGACCAGCAAGATCAAGACG |
| q18SrRNA_F | CTACGTCCCTGCCCTTTGTACA |
| q18SrRNA_R | ACACTTCACCGGACCATTCAA |
| qOstubulin_F | GGAGTCACATGCTGCCTAAGGTT |
| qOstubulin_R | TCACTGCCAGCTTACGGAGG |
| qOsPAL06_F | CGTCCGCATCAACACACTCC |
| qOsPAL06_R | CGTTCACCTTGCTGCCATCT |
| qOsXa5_F | GTTGCTGTTCCTACCCCCCA |
| qOsXa5_R | CAACGCTGAATCCACAACCA |
