# Supplementary material for: The Maize NBS-LRR Gene ZmNBS25 Enhances Disease Resistance in Rice and Arabidopsis
Source: Front Plant Sci. 2018 Jul 17;9:1033. doi: 10.3389/fpls.2018.01033 (PMC6056734; doi:10.3389/fpls.2018.01033)
Supplement: TABLE S2 — Disease resistance proteins in various plant species. [file Table_2.DOCX]

Table S2 Information of resistance proteins in other plants.

| Name | GenBank | Species |
| --- | --- | --- |
| AtRPM1 | CAA61131.1 | *Arabidopsis thaliana* |
| ZmRp1 | AAP81261.1 | *Zea mays* |
| OsXA1 | BAA25068.1 | *Oryza sativa indica Group* |
| SbPc-A | ACB72454.1 | *Sorghum bicolor* |
| TaLr10 | ADM65840.1 | *Triticum dicoccoides* |
| HvMla1 | AAG37356.1 | *Hordeum vulgare* |
| ZmRXO1 | AAX31149.1 | *Zea mays* |
| ZmRGA4 | NP_001147651.1 | *Zea mays* |
| HvSL8 | AJ507098 | *Hordeum vulgare* |
| ZmMRPR1 | NM_001112339.1 | *Zea mays* |
| SiRPM1-like | XM_004978992.1 | *Setaria italica* |
| OsYR5 | AF456245.1 | *Oryza sativa* |
| OsRGA5 | AGM61351.1 | *Oryza sativa Japonica Group* |
