# Supplementary material for: The Maize NBS-LRR Gene ZmNBS25 Enhances Disease Resistance in Rice and Arabidopsis
Source: Front Plant Sci. 2018 Jul 17;9:1033. doi: 10.3389/fpls.2018.01033 (PMC6056734; doi:10.3389/fpls.2018.01033)
Supplement: TABLE S4 — Statistical analyses of genes relative expression levels for Figure 5E. [file Table_4.DOCX]

Table S4 Statistical analyses of genes relative expression levels for Figure 5E

| Gene names | Treatment | Relative expression levels | | | *t*-test (P-value) | | | |
| --- | --- | --- | --- | --- | --- | --- | --- | --- |
|  |  | Rep1 | Rep2 | Rep3 | Col-0-Mock | Col-0-*Pst* DC3000 | *ZmNBS25*-OE1-Mock | ZmNBS25-OE1-*Pst* DC3000 |
| *AtEDS1* | Col-0-Mock | 1.030 | 0.968 | 1.002 |  | 0.001 | 0.002 | 0.000 |
|  | Col-0-*Pst* DC3000 | 4.056 | 3.524 | 3.064 | 0.001 |  | 0.744 | 0.001 |
|  | *ZmNBS25*-OE1-Mock | 4.193 | 4.048 | 2.921 | 0.002 | 0.744 |  | 0.003 |
|  | *ZmNBS25*-OE1-*Pst* DC3000 | 7.106 | 7.951 | 6.669 | 0.000 | 0.001 | 0.003 |  |
| *AtNDR1* | Col-0-Mock | 1.087 | 0.986 | 0.932 |  | 0.000 | 0.002 | 0.000 |
|  | Col-0-*Pst* DC3000 | 12.576 | 9.328 | 10.671 | 0.000 |  | 0.001 | 0.464 |
|  | *ZmNBS25*-OE1-Mock | 2.663 | 3.151 | 2.378 | 0.002 | 0.001 |  | 0.000 |
|  | *ZmNBS25*-OE1-*Pst* DC3000 | 11.767 | 10.650 | 12.957 | 0.000 | 0.464 | 0.000 |  |
| *AtPRS5* | Col-0-Mock | 0.794 | 0.998 | 1.263 |  | 0.268 | 0.916 | 0.098 |
|  | Col-0-*Pst* DC3000 | 1.135 | 1.483 | 1.128 | 0.268 |  | 0.218 | 0.607 |
|  | *ZmNBS25*-OE1-Mock | 0.771 | 1.014 | 1.207 | 0.916 | 0.218 |  | 0.069 |
|  | *ZmNBS25*-OE1-*Pst* DC3000 | 1.368 | 1.287 | 1.291 | 0.098 | 0.607 | 0.069 |  |
| *AtPR1* | Col-0-Mock | 0.969 | 1.030 | 1.002 |  | 0.002 | 0.002 | 0.002 |
|  | Col-0-*Pst* DC3000 | 1.735 | 2.171 | 2.047 | 0.002 |  | 0.504 | 0.005 |
|  | *ZmNBS25*-OE1-Mock | 2.412 | 2.055 | 1.919 | 0.002 | 0.504 |  | 0.006 |
|  | *ZmNBS25*-OE1-*Pst* DC3000 | 5.991 | 6.510 | 4.364 | 0.002 | 0.005 | 0.006 |  |
| *AtTAO1* | Col-0-Mock | 0.996 | 0.904 | 1.110 |  | 0.005 | 0.001 | 0.000 |
|  | Col-0-*Pst* DC3000 | 5.683 | 4.597 | 3.501 | 0.005 |  | 0.572 | 0.034 |
|  | *ZmNBS25*-OE1-Mock | 5.996 | 4.918 | 4.335 | 0.001 | 0.572 |  | 0.041 |
|  | *ZmNBS25*-OE1-*Pst* DC3000 | 6.186 | 7.300 | 7.065 | 0.000 | 0.034 | 0.041 |  |
| *AtPR5* | Col-0-Mock | 1.134 | 0.852 | 1.035 |  | 0.020 | 0.361 | 0.840 |
|  | Col-0-*Pst* DC3000 | 2.909 | 4.166 | 2.273 | 0.020 |  | 0.039 | 0.029 |
|  | *ZmNBS25*-OE1-Mock | 1.775 | 1.020 | 1.037 | 0.361 | 0.039 |  | 0.597 |
|  | *ZmNBS25*-OE1-*Pst* DC3000 | 1.580 | 0.950 | 0.672 | 0.840 | 0.029 | 0.597 |  |
|  |  |  |  |  |  |  |  |  |
